# Bitter melon extract inhibits breast cancer growth in preclinical model by inducing autophagic cell death

**DOI:** 10.18632/oncotarget.19887

**Published:** 2017-08-03

**Authors:** Naoshad Muhammad, Robert Steele, T. Scott Isbell, Nancy Philips, Ratna B. Ray

**Affiliations:** ^1^ Department of Pathology, Saint Louis University, St. Louis, Missouri, USA; ^2^ Cancer Center, Saint Louis University, St. Louis, Missouri, USA

**Keywords:** bitter melon, breast cancer cells, autophagy, LC3B, AMPK

## Abstract

Breast cancer is a major public health problem worldwide in women and current therapeutic strategies are not adequately effective for this deadly disease. We have previously shown the anti-proliferative activity of bitter melon extract (BME) in breast cancer cells. In this study, we observed that BME treatment induces autophagosome-bound Long chain 3 (LC3)-B and accumulates protein p62/SQSTM1 (p62) in breast cancer cells. Additionally, we observed that BME treatment in breast cancer cells increases phospho-AMPK expression and inhibits the mTOR/Akt signaling pathway. Subsequently, we demonstrated that BME feeding effectively inhibited breast cancer growth in syngeneic and xenograft mouse models. Further, we observed the increased p62 accumulation, induction of autophagy and apoptotic cell death in tumors from BME-fed animals. Taken together, our results demonstrate that BME treatment inhibits breast tumor growth, and this anti-tumor activity in breast cancer is, in part, mediated by induction of autophagy and modulation of the AMPK/mTOR pathway. The antitumor activity of BME by oral feeding in breast cancer models suggested the high potential for a clinical application.

## INTRODUCTION

Breast cancer is the predominant cancer among women in the world-wide and the leading cause of cancer-related mortality [1]. The American Cancer Society estimates approximately 300,000 women were diagnosed with breast cancer and among them ∼30,000 died from this deadly disease in 2016 (American Cancer Society: Breast cancer facts and figures 2015-2016). Although surgery and certain chemotherapy drugs reduce the risk of breast cancer recurrence in some women who are at risk of developing the metastatic disease or a recurrence, the adverse side effects limits their use [2]. Therefore, the use of preventive medicines is becoming increasingly important.

Natural products play a critical role in the discovery and the development of numerous drugs for the treatment of various types of deadly diseases including cancer [3, 4]. Bitter melon (*Momordica charantia*) is extensively cultivated in Asia, Africa, and South America, and is widely used in folk medicines to treat diabetes [5]. In our previous studies, we showed that bitter melon extract (BME) exerts a strong anti-proliferative effect by inducing caspase-dependent apoptosis in breast cancer cells [6], although the *in vivo* effect of BME was not examined in breast cancer model.

Autophagy is an evolutionally conserved catabolic process in all eukaryotic cells and contributes to organelle turnover, protein degradation, and differentiation [7, 8]. Growing evidence suggested that autophagy and apoptosis are interconnected positively or negatively in controlling cancer [7]. Recent studies implicated autophagy to be an expected and potential new approach against cancer [9]. Several natural products such as resveratrol, ivermectin, hernandezine, allspice and others have been shown to induce autophagic cell death in various types of cancer [10-14].

To our knowledge, this is the first study demonstrating that BME induces p62 accumulation and autophagic cell death by modulating AMPK/mTOR signaling pathway in breast cancer cells. We further demonstrated that BME feeding inhibited the tumor growth in different breast cancer mouse models, suggesting its potency is an attractive chemotherapeutic regimen against breast cancers.

## RESULTS

### BME induced autophagy in breast cancer cells

**Figure 1:**
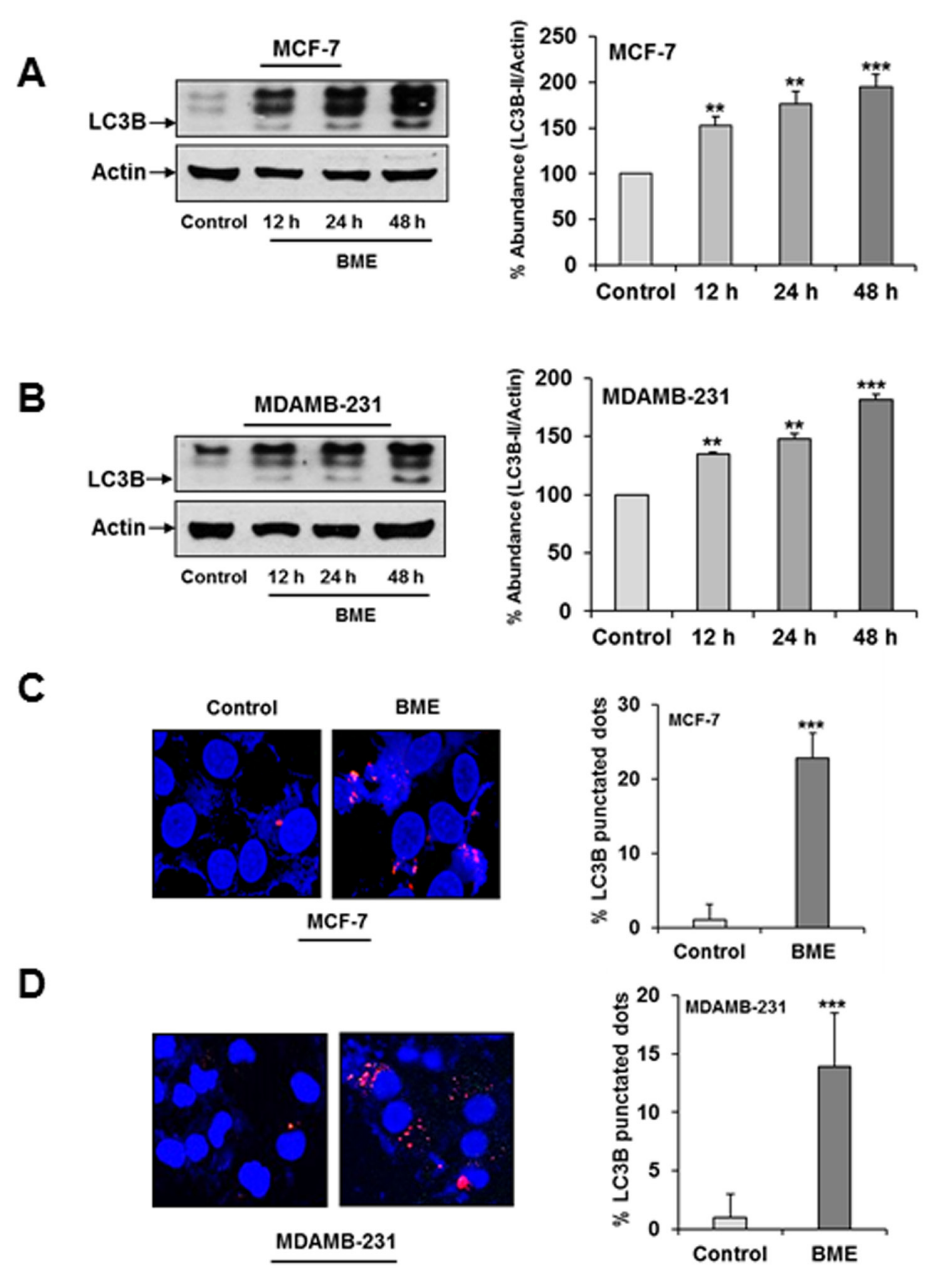
BME treatment induces autophagy in breast cancer cells **(A)** MCF-7 **(B)** MDAMB-231cells were treated with BME (2%, v/v) at indicated time points. Cell lysates were analyzed by Western blot for LC3B (16 KDa and 14 KDa) expression using specific antibody. The blot was reprobed with an antibody to actin (43 KDa) for comparison of protein load. Densitometry analyses was performed using Image J software and shown on the right. Data are represented as mean ± SD from three different experiments. Small bar indicates standard error (**, p<0.01, ***, p<0.001). **(C)** MCF-7 and **(D)** MDAMB-231 cells were treated with BME for 24 h, fixed, and permeabilized before viewing by confocal microscopy. Bar chart represents the quantitation of autophagic cells with LC3B puncta. Data are represented as mean ± SD. Small bar indicates standard error (***, p<0.001).

**Figure 2:**
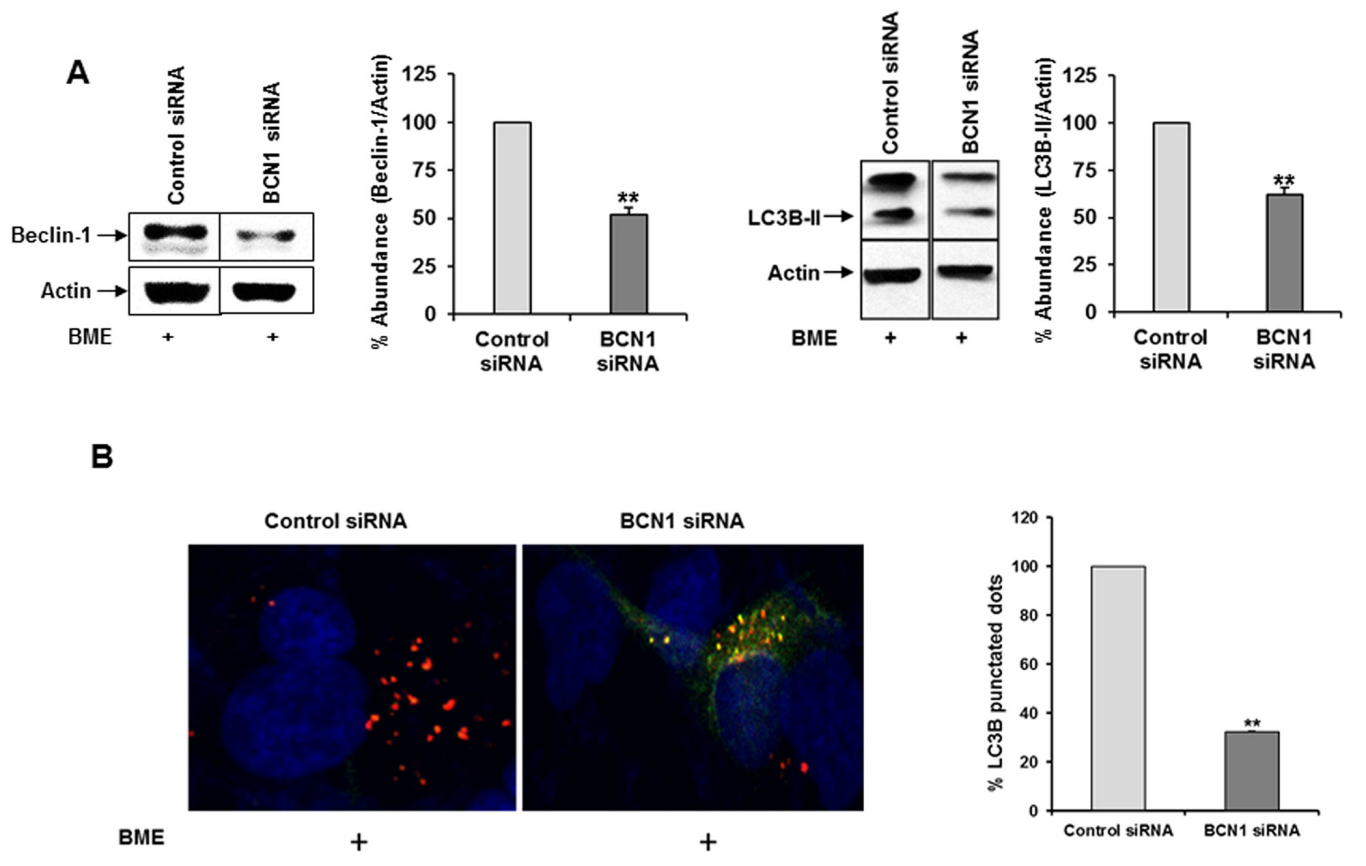
Knockdown of Beclin-1 suppresses BME induced autophagy in breast cancer cells **(A)** MDAMB-231 cells were transfected with control or Beclin-1 siRNA and then treated with BME (2%, v/v). Cell lysates were subjected to Western blot analysis using Beclin1 (60KDa) and LC3B antibodies. The blot was reprobed with an antibody to actin for comparison of protein load. Densitometry analyses was performed using Image J software and shown on the right. Data are represented as mean ± SD from three different experiments. Small bar indicates standard error (**, p<0.01). **(B)** Control or Beclin-1 siRNA transfected and BME treated MDAMB-231 cells were fixed, and examined for LC3B puncta as described in Figure 1. The GFP (green) tag is acid-sensitive and suggesting no lysosomal fusion. Bar chart represents the quantitation of autophagic cells with LC3B puncta. Data are represented as mean ± SD. Small bar indicates standard error (**, p<0.01).

We examined the in-depth mechanism of BME mediated breast cancer cell death. Increasing evidence suggested a critical role of drug-induced autophagy as anticancer therapy [15]. We examined the effect of BME on formation of the autophagosome membrane by detecting the conversion of LC3A to lipidated LC3B. BME treatment resulted in marked autophagy induction in a time-dependent manner in MCF-7 and MDAMB-231 cell lines as compared to untreated control cells (Figure 1 panels A and B). To corroborate BME-induced autophagy, the appearance of punctated dots of LC3B was examined by confocal microscopy. We observed that the formation of LC3B puncta were increased in both cell lines treated with BME (Figure 1 panels C and D), indicating that BME initiated the process of autophagy. Similar result was also obtained in ZR-75 breast cancer cells treated with BME (data not shown). To further verify that BME indeed induces autophagy, we knocked down Beclin-1, one of the key molecules in the autophagy pathway, using siRNA to Beclin-1. Our results showed that knockdown of Beclin-1 inhibited BME induced LC3 lipidation as compared to control siRNA treated cells (Figure 2, panel A). Further, formation of LC3B puncta was significantly reduced in Beclin-1 siRNA transfected BME treated MDAMB-231 cells (Figure 2, panel B). Together, these results suggested that BME treatment induces autophagy in breast cancer cells.

**Figure 3:**
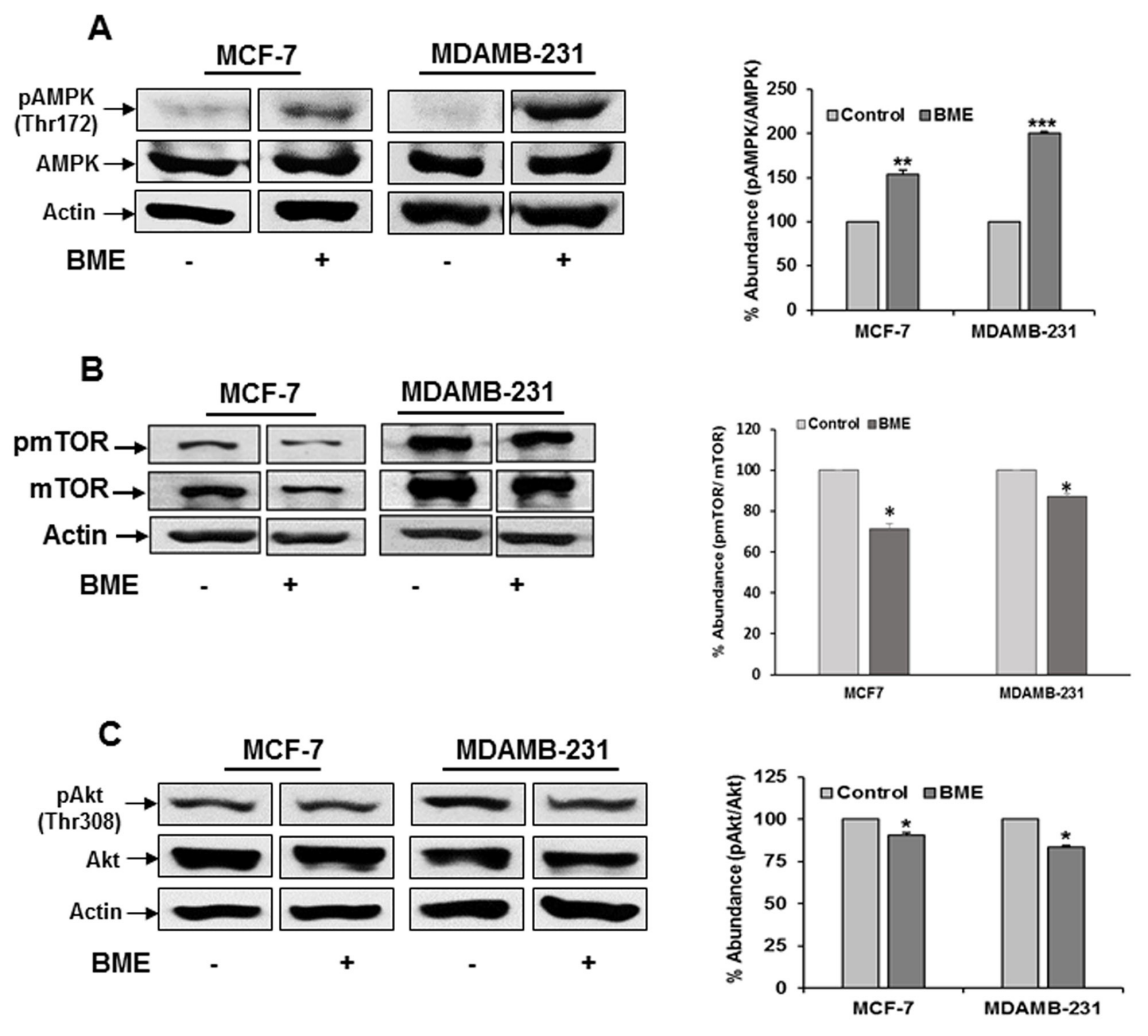
BME treatment modulates the AMPK/mTOR/Akt signaling pathway in breast cancer cells Cells were treated with BME (2%, v/v) for 48h, and cell lysates were analyzed by Western blot for **(A)** pAMPK/AMPK (62 KDa), **(B)** p-mTOR/mTOR (289 KDa) and **(C)** pAkt/Akt (60KDa) expression using specific antibodies. The blot was reprobed with an antibody to actin for comparison of protein load. Densitometry analyses was performed from three experiments using Image J software and shown on the right. Data are represented as mean ± SD. Small bar indicates standard error (*, p<0.05, **, p<0.01, ***, p<0.001).

BME treatment enhanced autophagy through the AMP-activated protein kinase (AMPK)-dependent inhibition of mTOR pathway. Recent studies have indicated that modulation of the AMPK/mTOR pathway is associated with the triggering of autophagy in cancer cells [10, 12]. We next investigated whether AMPK/mTOR pathway was involved in BME induced autophagy in breast cancer cell lines. AMPK promotes autophagy by suppressing mTOR [16, 17]. We examined the activation of AMPK in BME treated breast cancer cell lines. We observed that BME activates pAMPK^Thr172^ while total AMPK levels did not change significantly (Figure 3, panel A). Our results also demonstrated that BME treatment inhibits p-mTOR^Ser2448^ expression in MCF-7 and MDAMB-231 cells (Figure 3, panel B). However, total mTOR expression was unaltered. The Akt/mTOR pathway plays a critical role in the regulation of autophagy. We observed inhibition of pAkt^Thr308^ in both MCF-7 and MDAMB-231 cell lines following BME treatment (Figure 3, panel C). Together, these results indicated that AMPK/mTOR/Akt signaling pathway plays a crucial role in BME-induced autophagy in breast cancer cells.

### BME treatment accumulates p62 in breast cancer cells

**Figure 4:**
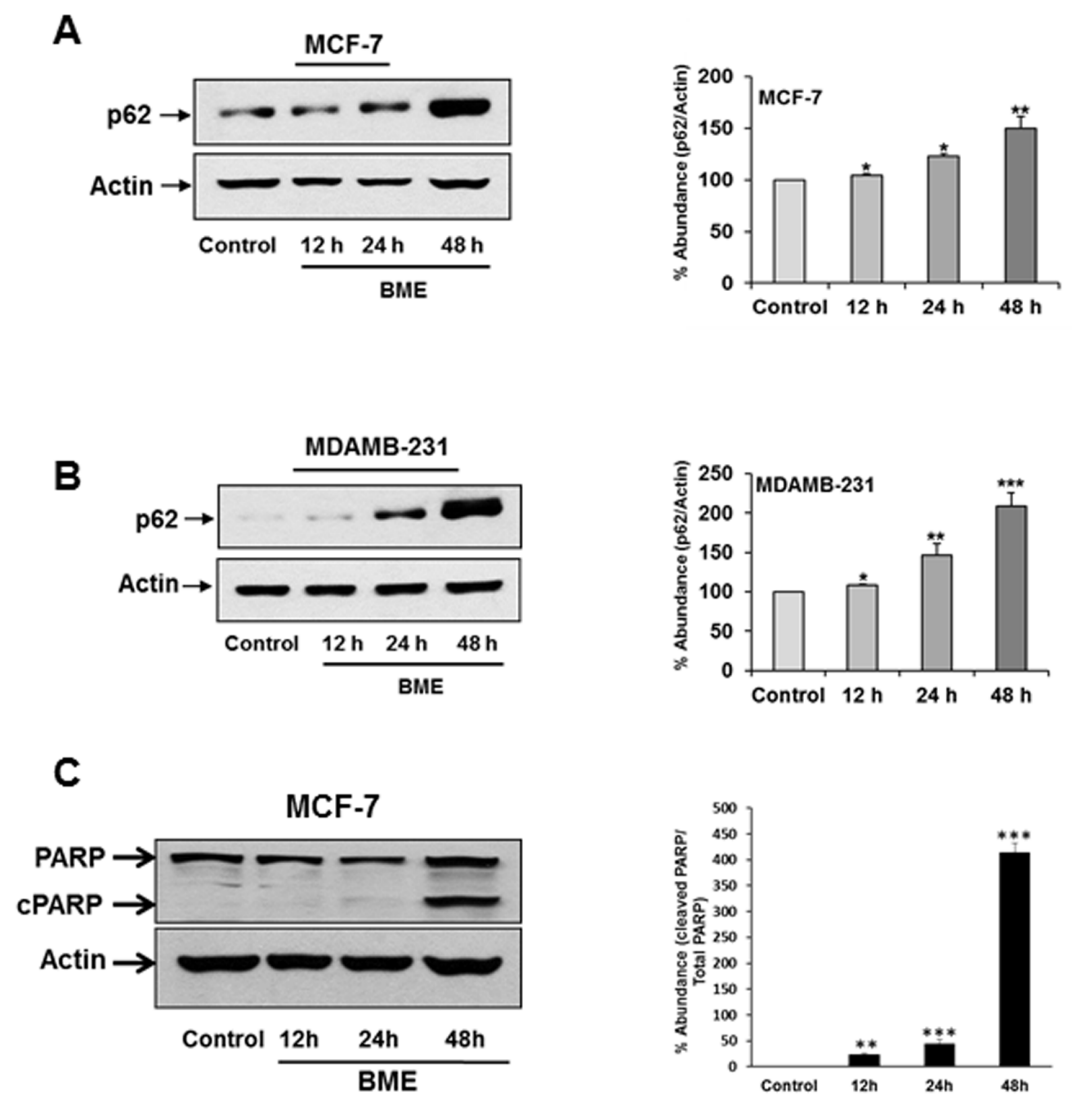
BME treatment accumulates p62 in breast cancer cells **(A)** MCF-7 **(B)** MDAMB-231cells were treated with BME at indicated time points. Cell lysates were analyzed by Western blot for p62 (62KDa) expression using specific antibody. **(C)** MCF-7 cell lysates were analyzed by Western blot for PARP expression using specific antibody. Total (116 KDa) and cleaved PARP (cPARP-86KDa) was shown. The blot was reprobed with an antibody to actin for comparison of protein load. Densitometry analyses was performed using Image J software from at least three experiments and shown at the right. Data are represented as mean ± SD. Small bar indicates standard error (*, p<0.05, **, p<0.01, ***, p<0.001).

The role of protein p62/SQSTM1 (p62) in the regulation of cancer is emerging. Several reports suggested that autophagy induction triggered elimination of p62 [18]. To further examine the signaling pathway that mediated the autophagic cell death in breast cancer cells by BME treatment, we investigated the expression level of p62 by Western blot analysis. Surprisingly, we observed that BME treatment accumulates p62 expression in a time-dependent manner in MCF-7 and MDAMB-231 cell lines as compared to untreated cell line (Figure 4, panels A and B). Resveratol treatment in chronic myelogenous leukemia cells displayed p62-dependent induction of autophagy by accumulating p62 [10]. p62 accumulation has also been correlated with caspase mediated apoptotic cell death [19]. We also observed PARP cleavage in BME treated MCF-7 breast cancer cells (Figure 4, panel C) as discussed previously [6]. A similar result was observed in MDAMB-231 breast cancer cells treated with BME (data not shown). Therefore, it is possible that BME mediated p62 accumulation correlates with apoptotic cell death.

### BME feeding inhibited tumor growth in different breast cancer mouse models

**Figure 5:**
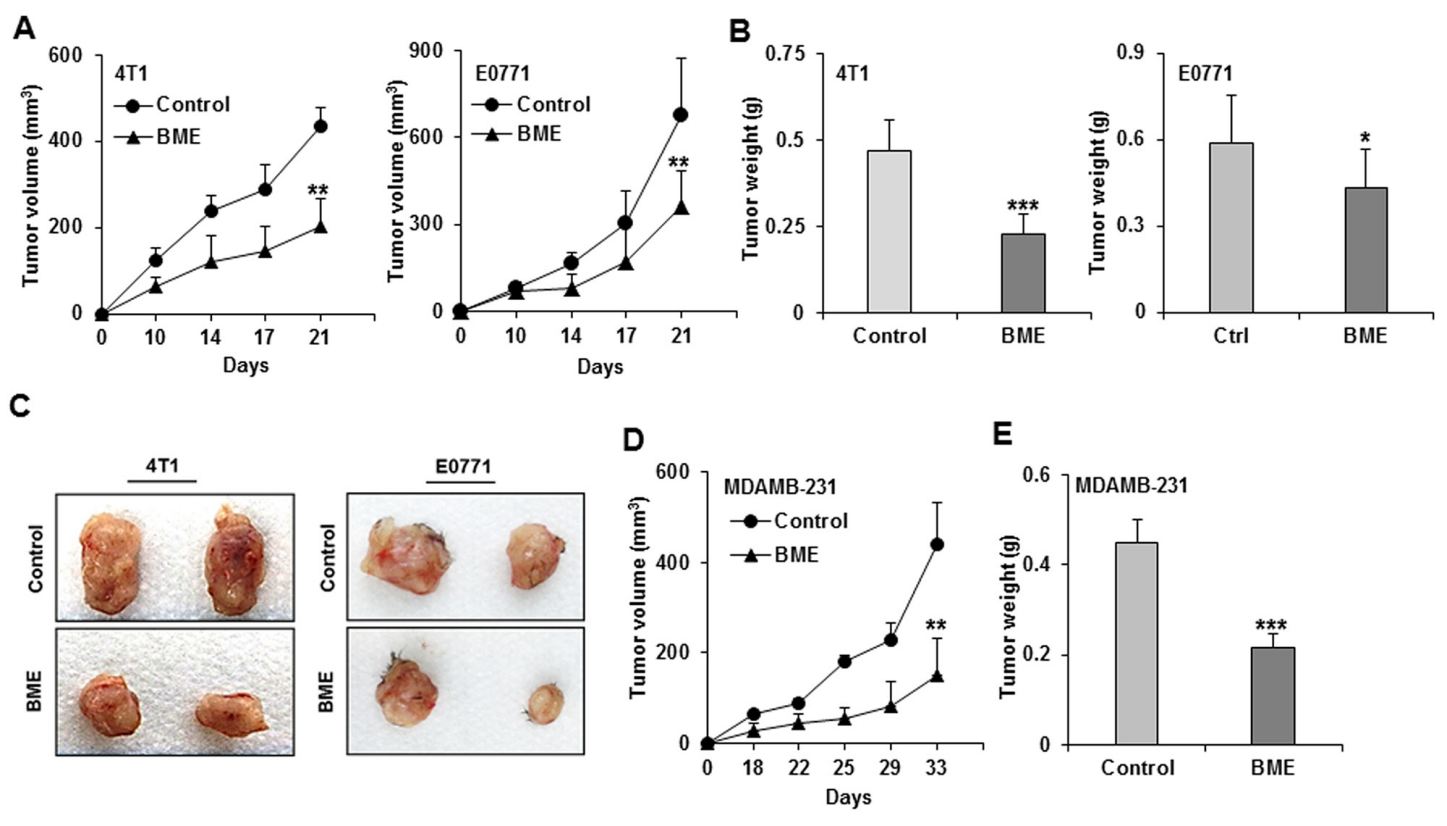
BME feeding inhibits tumor growth in preclinical models for breast cancer 4T1, E0771 and MDAMB-231 cells were implanted on the fifth mammary fad pad into the Balb/c, C57BL/6 and nude mice, respectively. Mice were randomized into two groups, and water (control) or BME in drinking water (experimental) was fed for entire duration of the experiment. **(A)** Tumor initiation and progression in 4T1 cells injected mice and E0771 cells injected mice were monitored as indicated time points and presented as a mean. **(B)** Tumor weight 4T1 and E0771 after scarification of mice and presented as a mean. **(C)** Representative tumors dissected from control and BME-fed mice 4T1 and E0771. **(D)** Tumor initiation and progression in MDAMB-231 cells injected mice were monitored as indicated time points and presented as a mean. **(E)** Tumor weight after scarification of mice and presented as a mean. Small bar indicates standard error (*, p<0.05, **, p<0.01, ***, p<0.001).

We have previously reported that BME inhibits the cell proliferation in breast cancer cells *in vitro* [6]. We here evaluated the efficacy of BME treatment in different breast cancer mouse models. We initially dosed BME at different concentration with drinking water in wild type C57/BL6 mice for 15 days and were unable to detect any toxicity. We chose to use 30% v/v dose for the *in vivo* study. The syngeneic breast cancer models were used using 4T1 and E0771 (mouse origin) cells, and implanted them into the mammary fat pad of Balb/c and C57BL/6 mice, respectively. Mice were divided into two groups. One group received BME (30% v/v, 600 mg/mouse/day) in their drinking water starting the day of tumor cell implantation. Other group received water as a control. We changed the water/BME every alternate day, and mice were regularly monitored. Fluid consumption was similar in both groups. Tumor volumes were significantly reduced in BME treated groups as comparison to control group in both mouse models (Figure 5, panel A). We also observed a significant reduction in the tumor weight of BME-fed mice as compared to control group (Figure 5, panel B). Macroscopically, the size of tumors was much larger in the control group than that of BME-fed mice. Representative images were shown from 4T1 and E0771 mouse models (Figure 5, panel C). Further, we wanted to verify whether BME treatment could inhibit tumor growth in human breast cancer cells using a xenograft model. For this, we implanted MDAMB-231 (human origin) cells into the mammary fat pad of nude mice. Mice were divided into two groups and treated as described above. BME-fed mice displayed a significant reduction in their tumor growth as compared to control group (Figure 5, panel D). We also observed a significant decrease in the tumor weight of these mice treated with BME as compared to control mice (Figure 5, panel E). Further to evaluate the effects of BME treatment on glucose metabolism, blood was collected from control and BME treated mice at the end of the study and processed for serum glucose measurement using routine methodologies in the clinical chemistry laboratory. Interestingly, we observed no statistically significant difference (data not shown) in post-prandial serum glucose concentrations between control and BME treated mice, suggesting that the dose we used in this study did not have a hypoglycemic effect. These results strongly demonstrated that BME treatment reduces the breast tumor growth in preclinical models.

### BME treatment induced autophagy in breast cancer mouse models

**Figure 6:**
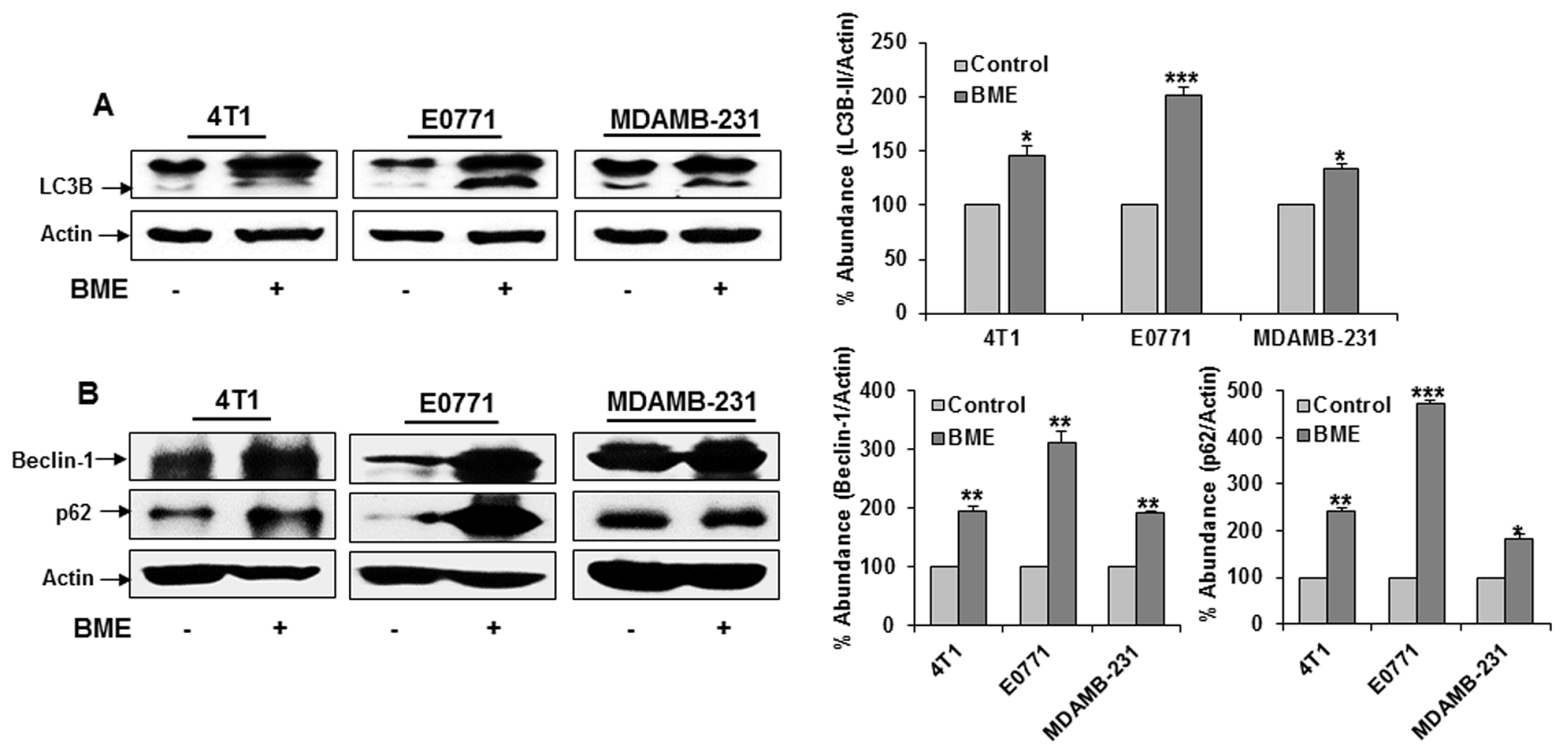
BME feeding induces autophagy in breast cancer mouse models Tumor lysates were prepared from tumors after sacrificing the mice. **(A)** Western blot analysis of LC3B in tumor tissues isolated from both control and BME-fed mice. The blot was reprobed with an antibody to actin for comparison of protein load. Densitometry analyses of these proteins were done by using Image J software and shown on the right. Data are represented as mean ± SD. **(B)** Western blot analysis of Beclin-1 and p62 in tumor tissues isolated from both control and experimental mice. The blot was reprobed with an antibody to actin for comparison of protein load. Densitometry analyses of these proteins were done by using Image J software and shown on the right. Data are represented as mean ± SD. Small bar indicates standard error (*, p<0.05, **, p<0.01, ***, p<0.001).

**Figure 7:**
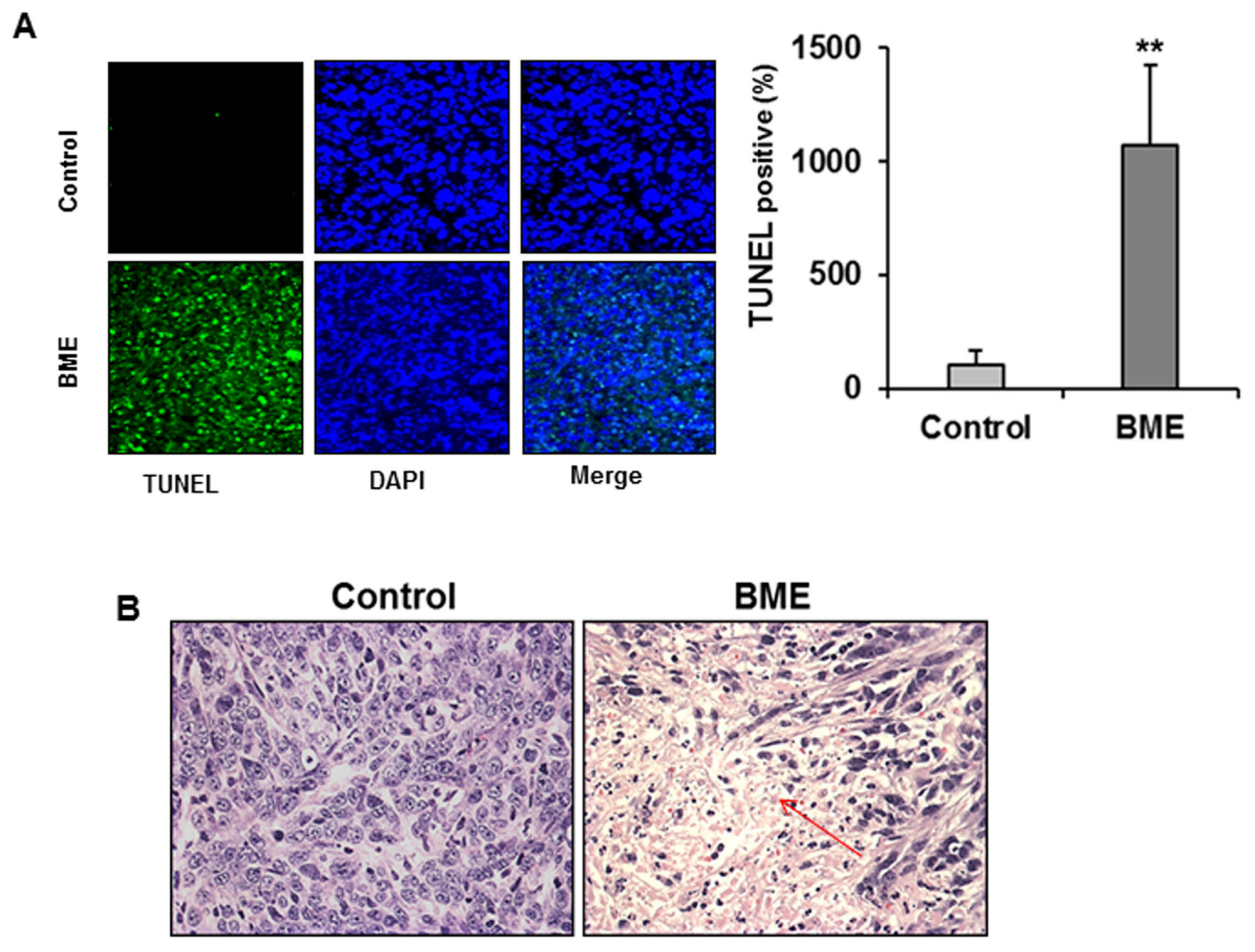
BME feeding induces apoptosis in breast cancer mouse model **(A)** Tumors from control or BME-fed mice were embedded in paraffin and TUNEL assay was performed from tissue sections. A reprehensive image has been shown from control and experimental groups. Quantitation of % TUNEL positive nuclei from control or BME-fed mice was performed blindly from at least ten sections and shown as a mean. Small bar indicates standard error (**, p<0.01). **(B)** A representative tumor section from control or BME-fed mice stained with hematoxylin and eosin (magnification, 40×) are shown. Arrows indicate apoptotic area.

We next verified BME induced autophagy in the tumor samples. Tumor lysates from BME-fed or control mice was examined for autophagy marker, LC3B. We observed LC3B lipidation in tumors from mice treated BME (Figure 6, panel A). Further, we observed that BME treatment in mice increased the expression of Beclin-1 in experimental group as compared to that of control mice (Figure 6, panel B). In addition, an accumulation of p62 in tumor lysates from BME-fed mice was noted (Figure 6, panel B). These results suggested that BME treatment inhibits tumor growth *in vivo* by inducing autophagy. Since we have observed PARP cleavage in BME treated breast cancer cells (6), we examined whether BME feeding induces apoptosis *in vivo*. The result of TUNEL assay demonstrated that BME treatment increased the number of TUNEL positive cells in BME-fed tumors as compared to control tumors (Figure 7, panel A). Further, H&E staining from tumor section suggested the induction of apoptosis in BME-fed mice, which was reflected with the tumor volume (Figure 7, panel B).

## DISCUSSION

Increasing evidence suggested that natural products play a promising and potential role in the development of novel chemotherapeutics for the treatment of cancers [14, 20]. We have reported previously the anti-proliferative and anticancer activity of BME involved various signaling mechanisms such as the induction of apoptosis, cell cycle arrest and suppression of c-Met signaling in several cancer models [21, 22]. However, the underlying mechanisms of the growth inhibitory effects of BME in breast cancer cells remains poorly understood. In this study, we demonstratedthat BME induced autophagy in breast cancer cells. We also demonstrated that BME treatment reduces breast tumor growth in different mouse models. The antitumor activity of BME by oral feeding in breast cancer models suggested the potential for a clinical application. BME feeding alone showed a significant antitumor activity consistently. Co-treatment with low dose of a chemotherapeutic drug may enhance anti-tumor activity and will be investigated in our future studies.

Autophagy serves a dual role as a mechanism of tumor suppression and promotion of cancer growth [23]. Autophagy functions as an anti-tumor mechanism by eliminating damaged organelles/proteins and restraining cell growth and genomic instability. A growing body of evidence has delineated that several natural products induce autophagy as an anticancer therapy, suggesting a novel therapeutic target whose modulation presents new opportunities for cancer treatment [14]. To date, this is the first study to our knowledge demonstrating that BME treatment induces autophagy in breast cancer cells *in vitro* and preclinical models *in vivo*. We observed BME-mediated activation of AMPK in breast cancer cells. AMPK, an important sensor of intracellular energy levels, maintains normal energy balance by regulating cellular metabolisms in an ATP/AMP ratio-dependent manner [24]. Bitter melon treatment induces autophagic cell death in pancreatic carcinoma cells [25]. AMPK has been implicated for mechanistic modulation of autophagy by inhibiting the mTOR signaling pathway.

Our data demonstrated that the induction of autophagy by BME is related to the inhibition of mTOR/Akt signaling. BME treatment decreases mTOR activity through downregulation of phosphorylated form of mTOR. The serine/threonine kinase mTOR has become a striking therapeutic target for the treatment of cancer and various studies revealed that mTOR kinase negatively regulates autophagy. mTOR regulates autophagy through various different molecular mechanisms [26, 27]. Our findings indicate that BME-induced autophagy in breast cancer cells involves an inhibition of the mTOR pathway.

We have observed both apoptosis and autophagy following the treatment of BME in breast cancer cells and preclinical models. Recent study reported that Paris saponins induce anticancer activity in breast cancer cells by inducing apoptosis and autophagy [28]. Akt/mTOR signaling pathway is involved in many cellular functions including apoptosis. The function of p62 in cancer is still emerging. p62 is plays an important role in autophagy, tumor growth regulation and apoptosis [10, 18, 19]. We observed accumulation of p62 in BME treated breast cancer cells and also in tumors. We have shown previously that BME treatment induces apoptosis cell death in breast cancer cells [6]. Therefore, it is important to consider when autophagy signaling molecules are targeted as a cancer therapeutic candidate. In-depth understating of how BME induces apoptosis and autophagy require further studies.

We observed that in 4T1 and MDAMB-231 mouse models, BME treatment responded better in tumor regression as compared to E0771 mouse model. Both 4T1 and MDAMB-231 cells are triple negative breast cancer cells, whereas E0771 is an ER positive breast cancer cell line. However, we did not observe differences in cell death in MCF-7 (ER positive) and MDA-MB-231 cells *in vitro*. We also did not observe change in serum glucose level in BME-fed group as compared to control group and no toxicity was observed in BME-fed mice. There are several components identified as active compounds for BME with limited follow-up studies. We identified Cholesteryl β-D-glucopyranoside as an active component of BME in *in vitro* study (unpublished data). However, efficacy of this compound is not very strong. In fact, much of the evidence in cancer chemoprevention suggests that bitter melon crude extract (mimicking the whole fruit components) has a stronger effect as compared to fractionated active components. A similar result was reported from black berry extract [29]. We should mention that AMPK activation is much higher in MDAMB-231 cells as compared to MCF-7 cells following BME treatment.

**Figure 8:**
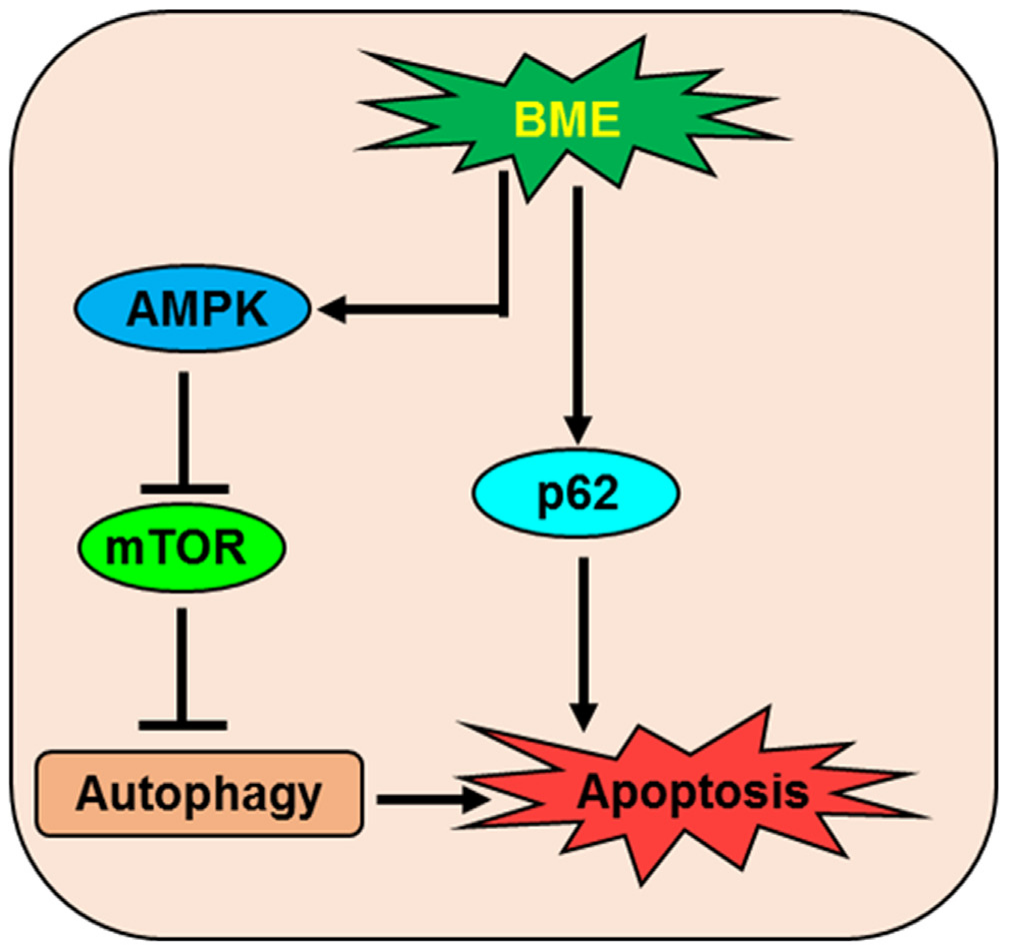
Schematic diagram showing the action of BME in the induction of autophagic cell death in breast cancer

In summary, we have demonstrated that BME induces autophagic cell death in breast cancer cells, both *in vitro* and *in vivo*, through the LC3B/AMPK/mTOR signaling pathways (Figure 8). Moreover, high efficacy of BME in triple negative breast cancer preclinical models strongly indicates its translational potential.

## MATERIALS AND METHODS

### Cells, transfection and BME preparation

Breast cancer cell lines (MCF-7 and MDAMB-231) were purchased from the American Type Culture Collection (ATCC) and maintained in RPMI-1640 or DMEM (Sigma) medium supplemented with 10% fetal bovine serum (FBS), and 1% penicillin/streptomycin in a humidified CO_2_ incubator. E0771 cells were obtained from Dr. Rong Xiang (Scripps Institute) and maintained in DMEM containing 10% fetal bovine serum. 4T1 cells were kind gift from Dr. David Piwnica-Worms at Washington University, St Louis and maintained in RPMI 1640 medium containing 15% fetal bovine serum. For stable cell line preparation, MCF-7 and MDAMB-231 cells were stably transfected with a plasmid expressing mCherry-EGFP-LC3 (kind gift of Kaustubh Datta). For this, approximately 60% confluent cells were seeded into 35 mm plates and transfected with 2 μg of mCherry-EGFP-LC3 with Jet prime reagent. Cells were selected with puromycin (1 mg/ml) and GFP-LC3-positive clones were pooled. MDAMB-231 cells were transfected with control (scrambled) or Beclin-1 siRNA (Santa Cruz Biotechnology) using Jet prime reagent. After 24 h of transfection, cells were treated with BME and cell lysates were prepared. BME was prepared from the Chinese variety of young bitter melons (raw and green) as discussed previously [6]. Briefly, BME was extracted from bitter melon without seeds using a household juicer, centrifuged at 15000 x g at 4°C for 30 min, and stored at -80 °C until used for *in vitro* and *in vivo* studies. We have initially used different doses of BME in breast cancer cells (*in vitro* studies) and normal mammary epithelial cells [6]. In this study, we used breast cancer cells treated with 2% BME (v/v) at different time points as described in the figure legends.

### Immunofluorescence

MCF-7 and MDAMB-231 cells stably expressing LC3 were seeded at 3x10^5^ in 35 mm plates and treated with BME for 24 h. Next, cells were washed twice with PBS, fixed and stained with DAPI. Images were taken using the Olympus FV1000 confocal system.

### Western blot analysis and antibodies

Cell lysates were prepared from adherent control and BME treated cells and analyzed by SDS-PAGE. Proteins were transferred onto 0.45 μM nitrocellulose membrane (Bio-Rad). Membranes were blocked using 5% low fat dry milk in TBST and probed with the respective primary antibodies. Proteins were detected using ECL Western Blotting Substrate (Thermo Scientific) and exposed to autoradiography. Protein loading was normalized using antibody to β-actin. The following antibodies were used in this study: pmTOR, mTOR, pAMPK, AMPK, pAkt, Akt and LC3B (Cell Signaling Technologies), Beclin-1 and β-actin (Santa Cruz Biotechnology), and p62 (Abnova). Primary antibodies were used 1:1000 dilution and secondary antibody was used in 1:7000 dilutions.

### *In vivo* studies

Animal experiments were performed according to the NIH guidelines, following a protocol approved by the Institutional Animal Care and Use Committee (IACUC) of Saint Louis University. Nude, Balb/c and C57BL/6 mice (6 week old females) were purchased from Charles River. Mice were housed in a specific pathogen free animal facility at the Saint Louis University. Cells (MDAMB-231, 4T1 and E0771) were suspended in 100 μl serum free medium and injected into the mammary fat pad. Mice were randomized into two groups, one group of mice received standard drinking water (control group) and the other group received BME (30% v/v, 600 mg/mouse/day) in the drinking water (experimental group) till the completion of study. Tumor volume was measured using digital caliper and tumor volume was calculated according to the formula L×W^2^×0.5 (L = length; W = width; all parameters in millimeters). After sacrificing, a portion of the tumor was snap-frozen and stored at -80 °C for biochemical and histopathological analyses. Serum was collected to measure the serum glucose level.

### TUNEL assay

Apoptosis in tumors was examined by TUNEL assay using the FragEL DNA Fragmentation detection Kit (BioRad), following manufacturer’s protocol. Quantitation of TUNEL positive nuclei from control or BME-fed mice was performed blindly from at least ten sections. Slides were scanned on an Olympus VS120 (Olympus Scientific Solutions Americas, Waltham, MA) dedicated slide scanner at 20x objective magnification. DAPI and DAPI+FITC signals in nuclei were quantitated using CellSens Dimension (Olympus Scientific Solutions Americas, Waltham, MA).

### Statistical analysis

Results were expressed as the mean ± standard deviation (SD), and statistical analyses were performed using a two-tailed paired or unpaired Student’s t test in GraphPad Prism 6 (GraphPad, La Jolla, CA). A *p* value of <0.05 was considered statistically significant.

## Abbreviations

BME: bitter melon extract
(LC3)-B: Long chain 3 (LC3)-B
AMPK: AMP-activated protein kinase
